# Automated Electrocardiogram Analysis Identifies Novel Predictors of Ventricular Arrhythmias in Brugada Syndrome

**DOI:** 10.3389/fcvm.2020.618254

**Published:** 2021-01-14

**Authors:** Gary Tse, Sharen Lee, Andrew Li, Dong Chang, Guangping Li, Jiandong Zhou, Tong Liu, Qingpeng Zhang

**Affiliations:** ^1^Tianjin Key Laboratory of Ionic-Molecular Function of Cardiovascular Disease, Department of Cardiology, Tianjin Institute of Cardiology, Second Hospital of Tianjin Medical University, Tianjin, China; ^2^Laboratory of Cardiovascular Physiology, Faculty of Medicine, Li Ka Shing Institute of Health Sciences, The Chinese University of Hong Kong, Hong Kong, China; ^3^Faculty of Science, University of Calgary, Calgary, AB, Canada; ^4^Xiamen Cardiovascular Hospital, Xiamen University, Xiamen, China; ^5^School of Data Science, City University of Hong Kong, Hong Kong, China

**Keywords:** Brugada syndrome, automated ECG, risk stratification, depolarization, repolarization

## Abstract

**Background:** Patients suffering from Brugada syndrome (BrS) are at an increased risk of life-threatening ventricular arrhythmias. Whilst electrocardiographic (ECG) variables have been used for risk stratification with varying degrees of success, automated measurements have not been tested for their ability to predict adverse outcomes in BrS.

**Methods:** BrS patients presenting in a single tertiary center between 2000 and 2018 were analyzed retrospectively. ECG variables on vector magnitude, axis, amplitude and duration from all 12 leads were determined. The primary endpoint was spontaneous ventricular tachycardia/ventricular fibrillation (VT/VF) on follow-up.

**Results:** This study included 83 patients [93% male, median presenting age: 56 (41–66) years old, 45% type 1 pattern] with 12 developing the primary endpoint (median follow-up: 75 (Q1–Q3: 26–114 months). Cox regression showed that QRS frontal axis > 70.0 degrees, QRS horizontal axis > 57.5 degrees, R-wave amplitude (lead I) <0.67 mV, R-wave duration (lead III) > 50.0 ms, S-wave amplitude (lead I) < −0.144 mV, S-wave duration (lead aVL) > 35.5 ms, QRS duration (lead V3) > 96.5 ms, QRS area in lead I < 0.75 Ashman units, ST slope (lead I) > 31.5 deg, T-wave area (lead V1) < −3.05 Ashman units and PR interval (lead V2) > 157 ms were significant predictors. A weighted score based on dichotomized values provided good predictive performance (hazard ratio: 1.59, 95% confidence interval: 1.27–2.00, *P*-value<0.0001, area under the curve: 0.84).

**Conclusions:** Automated ECG analysis revealed novel risk markers in BrS. These markers should be validated in larger prospective studies.

## Introduction

Brugada syndrome (BrS), originally described in 1992, is an electrical disease that is associated with higher risks of life-threatening ventricular tachycardia (VT)/ventricular fibrillation (VF) and sudden cardiac death (SCD). Symptoms (1, 2), ECG markers (3) and invasive tests such as electrophysiological studies (4–6) have been used for risk stratification, but prediction remains difficult (7), especially in asymptomatic patients (8). In prior studies, ECG markers have been determined manually, but these measurements are limited by inter-observer variability and have subjective bias. By contrast, automated measurements have not been used for risk prediction, yet they may reveal useful information that is difficult to extract manually (9, 10). In this study, we extracted raw ECG data files, exported the automated measurements and tested the hypothesis a score system based on these variables can predict spontaneous VT/VF in a cohort of BrS patients.

## Methods

### Study Population

This retrospective study received Ethics approval from The Joint Chinese University of Hong Kong – New Territories East Cluster Clinical Research Ethics Committee and is based on datasets that have already been made available in an online repository (https://zenodo.org/record/3266172; https://zenodo.org/record/3266179; https://zenodo.org/record/3351892). The diagnosis of BrS is made based on the 2017 ACC/AHA/HRS Guideline (11), after reviewing documented patient history, and confirmed by analysis of all documented ECG by S.L. and G.T. Type 1 Brugada pattern is defined as a coved-shape ST segment with elevation of >2 mm followed by a negative T-wave, and type 2 pattern is defined as convex ST segment with >0.5 mm elevation followed by variable T-wave, resulting in a saddleback-shaped morphology (12). The study inclusion criteria were: (1) BrS diagnosis and (2) raw ECG data were available for automated ECG analysis.

### Baseline Characteristics and ECG Measurements

Clinical data was extracted from electronic health records. The following baseline clinical data were collected: (1) sex; (2) age of initial Brugada pattern presentation; (3) follow-up period; (4) type of Brugada pattern and presence of fever at initial presentation; (5) family history of BrS and VF/ SCD; (6) manifestation of syncope and if present, the number of episodes; (7) manifestation of VT/VF and if present, the number of episodes; (8) sodium channel blocker challenge test and results; (9) concomitant presence of other arrhythmia; (10) implantation of ICD. Patients presented with two or more episodes of VT/VF were defined to be of high VT/VF burden. Automatically measured parameters from ECG related to the P, Q, R, S and T-wave were extracted. The full list of variables is shown in Supplementary Table 1.

### Primary Outcome, Statistical Analysis, and Creation of a Score-Based System for Risk Prediction

The primary outcome was new occurrences of spontaneous VT/VF after diagnosis of BrS. The outcome was assessed by review of inpatient and outpatient case records. Cox regression was used to identify ECG variables that were significant predictors of the primary outcome. The following steps were undertaken to create a score system for risk stratification: (1) the variables related to Q, R, S and T waveforms that achieved *P*-values < 0.10 were identified, (2) related variables were discarded, (3) the location out of all 12 leads with the lowest *P*-values was selected, (4) optimum cut-off was calculated from receiver operating characteristic analysis, (5) each variable was dichotomized based on the cut-off, (6) calculation of beta coefficient and ORs for each dichotomized variable, (7) weight-adjusted score by proportion of beta coefficients and *P*-values.

## Results

**Figure 1:**
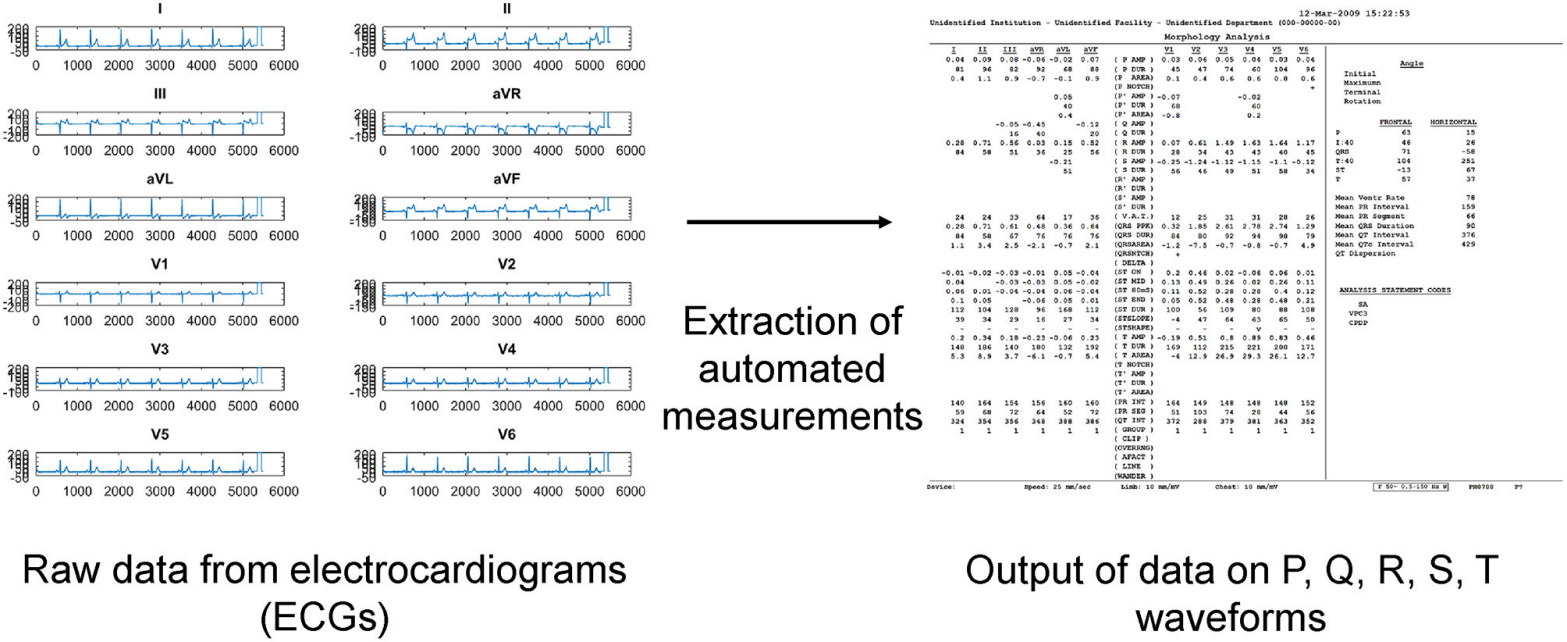
Extraction of automated electrocardiographic (ECG) variables from raw data files.

A total of 83 patients were included [93% male, median presenting age: 56 (41–66) years old] were included. The clinical characteristics of this cohort are shown in Table 1. The prevalence of an initial type 1 Brugada pattern on presentation was 45%. Twelve patients developed spontaneous VT/VF with a median follow-up of 74 (Q1–Q3: 26–114) months. Automated measurements of the ECG variables were extracted from the raw data (Figure 1).

**Table 1:** Baseline characteristics of the Brugada patients (*n* = 83) included in this study.

**Characteristics**	**Count**	**Hazard ratio (HR)^#^**	**95% CI**	***P*-value**	**Hazard ratio (HR)^∧^**	**95% CI**	***P*-value**
Female gender	6 (7)	1.10	0.14–8.50	0.931			
Age of Initial Presentation	56 (41–66)	0.99	0.95–1.02	0.455	0.98	0.95–1.02	0.383
Initial Type 1 BrP	37 (45)	3.64	1.08–12.30	**0.037**	3.02	0.91–10.04	0.072
Type 1 BrP	52 (63)	1.96	0.53–7.25	0.313	1.94	0.53–7.18	0.319
Evolution	29 (35)	0.46	0.12–1.73	0.251	0.54	0.15–1.99	0.355
Fever-induced type 1	11 (13)	1.49	0.33–6.85	0.607	1.50	0.33–6.86	0.599
FH BrS	3 (4)	2.03	0.25–16.24	0.503	3.14	0.41–24.33	0.273
Family History of VF/SCD	6 (7)	1.02	0.13–7.92	0.985	1.10	0.14–0.50	0.929
Syncope at initial presentation	29 (35)	5.24	1.05–26.20	**0.044**	4.72	0.95–23.39	**0.057**
Syncope at any point	43 (52)	5.62	1.22–25.94	**0.027**	4.66	1.02–21.29	**0.047**
# syncope	65 (83)	–	–	–	–	–	–
VT/VF at initial presentation	9 (11)	7.14	2.23–22.85	**0.001**	7.80	2.47–24.58	** <0.0001**
VT/VF at any point	16 (19)	–	–	–	–	–	–
High VT/VF Burden	6 (7)	18.96	5.69–63.13	** <0.0001**	19.38	6.25–60.14	** <0.0001**
Drug Challenge Performed	51 (61)	0.90	0.27–3.00	0.858	1.03	0.31–3.42	0.966
Drug Positive^*^	49 (96)	0.18	0.02–1.60	0.125	0.26	0.03–2.08	0.202
ICD	29 (35)	–	–	**–**	**–**	**–**	**–**
Other Arrhythmia	12 (14)	0.45	0.06–3.48	0.440	0.50	0.07–3.90	0.512

*Hazard ratios for predicting incident spontaneous VT/VF from Cox regression*.

* *Denominator only included patients undergoing testing. Variables with P <0.05 are shown in bold text*.

# *Breslow methods for ties*.

∧ *Parametric model with Weibull distribution*.

**Figure 2:**
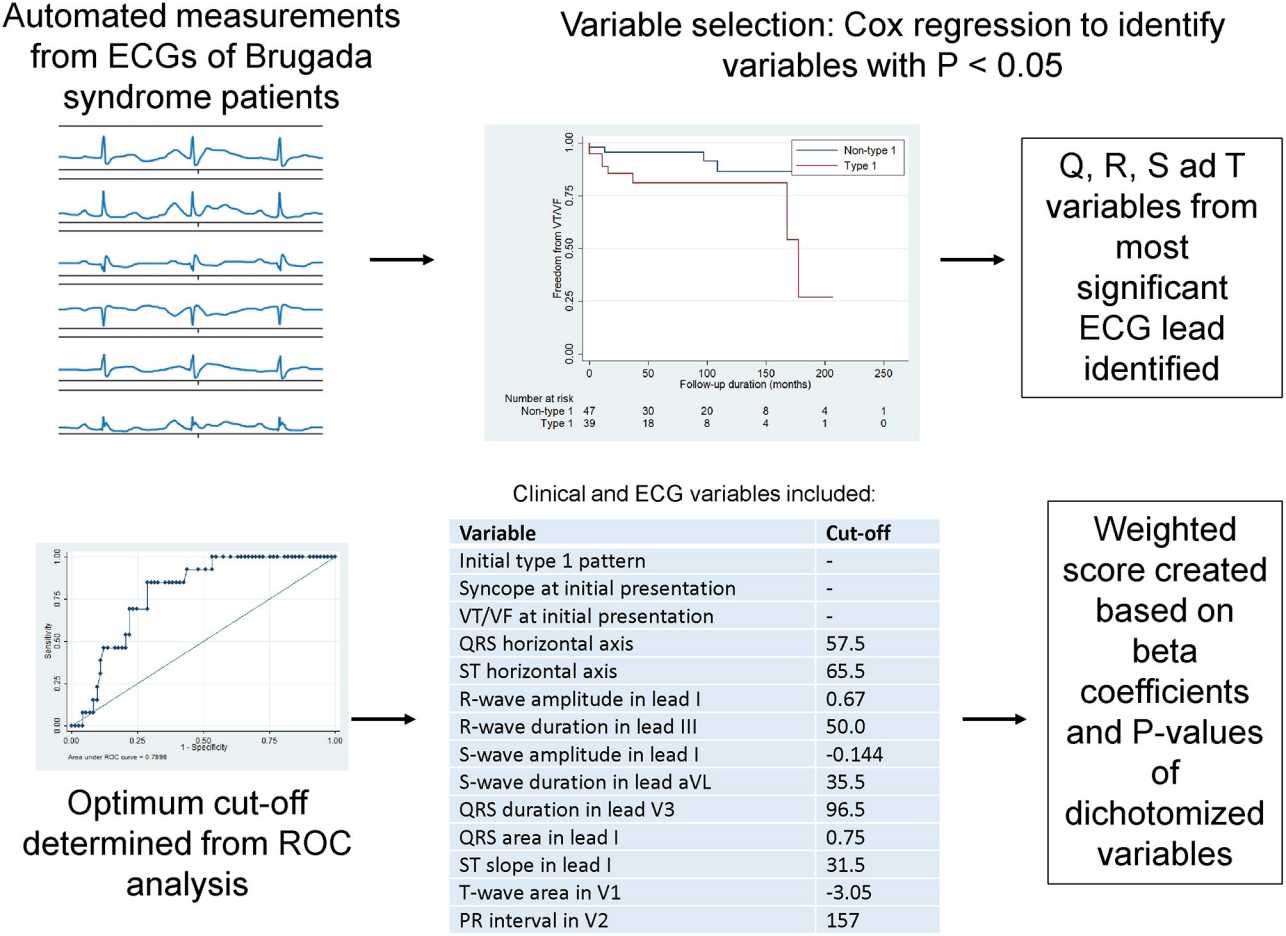
Steps for creating a weighted score system based on automated ECG measurements on ventricular depolarization and repolarization.

A weighted score system for risk stratification was created, as illustrated in Figure 2. Briefly, ECG variables related to Q, R, S and T waveforms, which achieved significance of *P*-values < 0.10 on Cox regression, were identified. Their median values (Q1–Q3) and hazard ratios (HR) with 95% confidence intervals (CIs) for classifying incident spontaneous VT/VF are shown in Table 2, whereas optimum cut-off values and area under the curve (AUC) from receiver operating characteristic (ROC) analysis are shown in **S2**. For each variable, the lead with the lowest *P*-value was selected. This selection process yielded 11 ECG variables: vector magnitude of the initial 40 ms of the transverse QRS signal, QRS horizontal axis, ST horizontal axis, R-wave amplitude in lead I, R-wave duration in lead III, S-wave amplitude in lead I, S-wave duration in lead aVL, QRS duration in lead V3, QRS area in lead aVL, ST slope in lead I, T-wave area in lead V1 and PR interval in lead V2. Vector magnitude of the initial 40 ms of the transverse QRS signal was not processed further as not all ECGs had this variable reported.

**Table 2:** Significant ECG variables of Q, R, S, and T waves for predicting incident spontaneous VT/VF from univariate Cox regression.

**Characteristics**	**Median (Q1–Q3)**	**Hazard ratio (HR)^#^**	**95% CI**	***P*-value**	**Hazard ratio (HR)^∧^**	**95% CI**	***P*-value**
Vector magnitude of the initial 40 ms transverse QRS signal (deg)	0.43 (0.30–0.70)	8.42	1.05–67.41	0.045	8.21	1.03–65.17	0.046
QRS horizontal axis (deg)	11 (−8 to 36)	1.01	1.001–1.012	0.024	1.01	1.001–1.012	0.030
ST wave horizontal axis (deg)	71 (53–83)	0.98	0.96–0.99	0.009	0.97	0.95–0.99	0.003
R-wave amplitude in lead I (mV)	0.50 (0.33–0.72)	0.06	0.004–0.86	0.038	0.12	0.01–1.35	0.086
R-wave duration in lead III (ms)	48 (28–60)	1.02	1.002–1.03	0.030	1.02	1.01–1.04	0.006
S-wave amplitude in lead I (mV)	−0.15 (−0.26 to −0.07)	0.006	0.0002–0.21	0.005	0.004	0.0001–0.13	0.002
S-wave duration in aVL (ms)	24 (0–48)	1.03	1.01–1.05	0.001	1.04	1.02–1.06	<0.0001
QRS duration in V3 (ms)	96 (88–104)	1.03	1.003–1.06	0.029	1.04	1.01–1.07	0.004
QRS area in lead I (ms.mV)	1.4 (−0.4 to 3.6)	0.67	0.54–0.84	0.001	0.69	0.56–0.84	<0.0001
ST slope in lead I (deg)	18 (9–33)	1.05	1.01–1.10	0.015	1.06	1.02–1.10	0.005
T-wave area in V1 (ms.mV)	−2.5 (−4.2 to −0.8)	0.82	0.73–0.93	0.002	0.80	0.70–0.90	<0.0001
PR interval in lead V2 (ms)	156 (144–176)	1.02	1.001–1.03	0.036	1.01	1.0002–1.03	0.046

*Median (Q1–Q3) and hazard ratios (HRs) with 95% confidence intervals (CIs) are presented. For each variable, only the lead with the lowest P-value across all 12 leads was shown*.

# *Breslow methods for ties*.

∧ *Parametric model with Weibull distribution*.

The remaining ECG variables were then dichotomized based on the optimum cut-off values from receiver operating characteristic (ROC) analysis. The dichotomized ECG variables were weighted based on the beta coefficients and *P*-values. After dichotomization, two variables lost significance for prediction (highlighted in red in Supplementary Table 3) and therefore the final score had a total of eight ECG and three clinical variables (Supplementary Table 4). A histogram plot for this *weighted score* is shown in Supplementary Figure 1. This weighted score provided good predictive performance when analyzed as a continuous variable [hazard ratio (HR): 1.59, 95% confidence interval (CI): 1.27–2.00, *P*-value < 0.0001, area under the curve (AUC): 0.84; Supplementary Figure 3] or a dichotomized variable (HR: 14.88, 95% CI: 3.99–55.50, *P*-value < 0.0001, AUC: 0.81) (Supplementary Table 5). A simplified algorithm was generated using decision tree learning for potential clinical application (AUC: 0.93, Supplementary Tables 6, 7; Supplementary Figure 3).

## Discussion

The main findings of this study is that (i) automated measurements from raw ECG data can be extracted and used for risk stratification, (ii) ST slope was identified as a novel risk marker, and (iii) a weighted score system based on QRS frontal axis, R-wave duration (lead III), S-wave duration (lead I), QRS duration (lead I) and ST slope (lead I) predicted incident spontaneous VT/VF with an AUC of 0.95.

Previously, investigators have commented that manual measurements may be susceptible to variations and errors (13). Indeed, accuracy and reproducibility of measurements made manually have not been examined (14). Our study provides the proof-of-concept that the axis of the QRS vector, depolarization and repolarization variables extracted automatically from raw ECG data can predict arrhythmic events with good fidelity. In BrS, both depolarization and repolarization abnormalities are posited to play important roles in ventricular arrhythmogenesis (15, 16). ECG indices related depolarization (13), such as QRS duration, QRS dispersion, R-wave and S-wave durations have been identified as useful predictors in this condition (3, 17–21). For Brugada syndrome, QRS vector magnitude was identified as a predictor of ventricular arrhythmias (22). In keeping with their findings, our study similarly demonstrated that the magnitude of initial 40 ms transverse QRS signal was borderline predictive of VT/VF (*P*-value = 0.051). However, the magnitude of the maximum transverse QRS vector or of its terminal portion were not significant predictors.

By contrast, repolarization abnormalities, as reflected by alterations in the ST segment, QT or T_peak_-T_end_ intervals, are also important arrhythmogenic substrates in BrS (23–26). Our novelty is the demonstration that the slope of ST segment is significantly associated with arrhythmic risk. Whilst the angle between the R' wave and the vertical line has been used to distinguish Brugada pattern from other causes with similar morphology, such as right bundle branch block (27), we are not aware of any previous study demonstrating the use of R or ST angles for risk stratification. Moreover, T_end_, and sometimes T_peak_, can be difficult to determine with a degree of certainty with different methods of determining its location (28). Recently, an automated algorithm calculated a global T_peak_ based on the root mean square average of T_peak_ from individual leads with a similar methodology for determining T_end_ (29). Whether these measurements provide more accurate risk stratification than manual measurements in BrS and other disease cohorts remain to be tested. Other than outcome prediction, other investigators have used automated ECG variables for disease detection and tracking (30). Future studies should examine whether serial changes in ECG variables can improve disease detection especially in type 2 Brugada subjects and be used to track disease progression in BrS.

From our predictive analysis, we generated a simple algorithm based on decision tree learning method for potential clinical application, as we have done so previously for other cohorts (31, 32). For Brugada syndrome, other decision tree-type algorithms have been proposed (33–35). These algorithms should be compared for their ability to predict arrhythmic outcomes. Previously, other groups have developed useful clinical risk scores for risk stratification in BrS. For example, Subramanian et al. proposed a score based on four variables: the presence of spontaneous type 1 pattern, QRS fragmentation in the inferior leads, S-wave upslope duration ≥0.8 and T_peak_-T_end_ intervals ≥ 100 ms with an excellent AUC of 0.95 (36). As not all of the above variables were obtained from the automated ECG outputs in our study. Future studies should develop novel algorithms to automatically identify the presence or absence of QRS fragmentation and to determine T_peak_-T_end_ intervals to allow comparisons of between the different risk scores.

## Limitations

Several limitations of our study should be noted. Firstly, the size of our cohort is relatively small. Our findings should be validated in larger prospective studies. Secondly, the majority of patients with detected VT/VVF events had ICDs implanted. Therefore, we cannot exclude ascertainment bias, where silent VT/VF events were missed in those without ICDs. Secondly, our extraction did not enable us to determine the T_peak_-T_end_ interval. Future work should focus on modifying existing algorithms to determine T_peak_ and T_end_, which would allow us to determine to extent to which repolarization abnormalities contribute to the arrhythmic substrate in BrS. Thirdly, the ECG predictors identified in this study may not be exclusive for BrS and may also be useful for risk stratification in other cardiovascular diseases such as myocardial infarction. This remains to be elucidated in future studies.

## Conclusions

Automated ECG measurements related to depolarization and repolarization are useful for risk stratification in BrS. These markers should be validated in larger prospective studies. If the predictability of automated measurements is verified, they have the potential to open the gate for the wide application of advanced machine learning models to facilitate risk stratification and clinical decision making in BrS and other diseases.

## Data Availability Statement

The raw data supporting the conclusions of this article are available in the datasets already made available in an online repository (https://zenodo.org/record/3266172; https://zenodo.org/record/3266179; https://zenodo.org/record/3351892).

## Ethics Statement

The studies involving human participants were reviewed and approved by the Joint Chinese University of Hong Kong – New Territories East Cluster Clinical Research Ethics Committee. Written informed consent for participation was not required for this study in accordance with the national legislation and the institutional requirements.

## Author Contributions

GT: study conception, study supervision, project planning, data interpretation, statistical analysis, manuscript drafting, and critical revision of manuscript. SL, DC, GL, AL, and JZ: data analysis, data interpretation, statistical analysis, manuscript drafting, and critical revision of manuscript. TL and QZ: data analysis, data interpretation, statistical analysis, study supervision, and critical revision of manuscript. All authors contributed to the article and approved the submitted version.

## Conflict of Interest

The authors declare that the research was conducted in the absence of any commercial or financial relationships that could be construed as a potential conflict of interest.
