# Supplementary material for: Automated Electrocardiogram Analysis Identifies Novel Predictors of Ventricular Arrhythmias in Brugada Syndrome
Source: Front Cardiovasc Med. 2021 Jan 14;7:618254. doi: 10.3389/fcvm.2020.618254 (PMC7840575; doi:10.3389/fcvm.2020.618254)
Supplement: Supplementary file 1 [file Data_Sheet_1.PDF]

Supplementary Appendix

Table S1. A list of ECG variables extracted from the raw data.

Automated ECG variables	Explanation
transqrsinitangle	Angle of initial 40 ms transverse QRS signal
transqrsinitmag	Magnitude of initial 40 ms transverse QRS signal
transqrsmaxangle	Angle of maximum transverse QRS vector
transqrsmaxmag	Magnitude of maximum transverse QRS vector
transqrstermangle	Angle of terminal 40 ms transverse QRS signal
transqrstermmag	Magnitude of terminal 40 ms transverse QRS signal
transqrscwrot	Direction of the vector rotation over the entire QRS complex
pfrontaxis	P-wave frontal axis
phorizaxis	P-wave horizontal axis
i40frontaxis	Initial 40 ms QRS complex frontal axis
i40horizaxis	Initial 40 ms QRS complex horizontal axis
qrsfrontaxis	QRS frontal axis
qrshorizaxis	QRS horizontal axis
t40frontaxis	Terminal 40 ms QRS complex frontal axis
t40horizaxis	Terminal 40 ms QRS complex horizontal axis
stfrontaxis	ST frontal axis
sthorizaxis	ST horizontal axis
tfrontaxis	T-wave frontal axis
thorizaxis	T-wave horizontal axis
atrialrate	Atrial rate
meanventrate	Ventricular rate
meanprint	Mean PR interval
meanprseg	Mean PR segment
meanqrsdur	Mean QRS duration
meanqtint	Mean QT interval
meanqtc	Mean QTc interval
qtintdispersion	QT interval dispersion
pampI	P-wave amplitude in lead I
pampII	P-wave amplitude in lead II
pampIII	P-wave amplitude in lead III
pampV1	P-wave amplitude in lead V1
pampV2	P-wave amplitude in lead V2
pampV3	P-wave amplitude in lead V3
pampV4	P-wave amplitude in lead V4
pampV5	P-wave amplitude in lead V5
pampV6	P-wave amplitude in lead V6
pampaVF	P-wave amplitude in lead aVF
pampaVL	P-wave amplitude in lead aVL
pampaVR	P-wave amplitude in lead aVR

pdurI	P-wave duration in lead I
pdurII	P-wave duration in lead II
pdurIII	P-wave duration in lead III
pdurV1	P-wave duration in lead V1
pdurV2	P-wave duration in lead V2
pdurV3	P-wave duration in lead V3
pdurV4	P-wave duration in lead V4
pdurV5	P-wave duration in lead V5
pdurV6	P-wave duration in lead V6
pduraVF	P-wave duration in lead aVF
pduraVL	P-wave duration in lead aVL
pduraVR	P-wave duration in lead aVR
pareaI	P-wave area in lead I
pareaII	P-wave area in lead II
pareaIII	P-wave area in lead III
pareaV1	P-wave area in lead V1
pareaV2	P-wave area in lead V2
pareaV3	P-wave area in lead V3
pareaV4	P-wave area in lead V4
pareaV5	P-wave area in lead V5
pareaV6	P-wave area in lead V6
pareaaVF	P-wave area in lead aVF
pareaaVL	P-wave area in lead aVL
pareaaVR	P-wave area in lead aVR
ppampI	P'-wave amplitude in lead I
ppampII	P'-wave amplitude in lead II
ppampIII	P'-wave amplitude in lead III
ppampV1	P'-wave amplitude in lead V1
ppampV2	P'-wave amplitude in lead V2
ppampV3	P'-wave amplitude in lead V3
ppampV4	P'-wave amplitude in lead V4
ppampV5	P'-wave amplitude in lead V5
ppampV6	P'-wave amplitude in lead V6
ppampaVF	P'-wave amplitude in lead aVF
ppampaVL	P'-wave amplitude in lead aVL
ppampaVR	P'-wave amplitude in lead aVR
ppdurI	P'-wave duration in lead I
ppdurII	P'-wave duration in lead II
ppdurIII	P'-wave duration in lead III
ppdurV1	P'-wave duration in lead V1
ppdurV2	P'-wave duration in lead V2
ppdurV3	P'-wave duration in lead V3
ppdurV4	P'-wave duration in lead V4
ppdurV5	P'-wave duration in lead V5
ppdurV6	P'-wave duration in lead V6
ppduraVF	P'-wave duration in lead aVF

ppduraVL	P'-wave duration in lead aVL
ppduraVR	P'-wave duration in lead aVR
ppareaI	P'-wave area in lead I
ppareaII	P'-wave area in lead II
ppareaIII	P'-wave area in lead III
ppareaV1	P'-wave area in lead V1
ppareaV2	P'-wave area in lead V2
ppareaV3	P'-wave area in lead V3
ppareaV4	P'-wave area in lead V4
ppareaV5	P'-wave area in lead V5
ppareaV6	P'-wave area in lead V6
ppareaaVF	P'-wave area in lead aVF
ppareaaVL	P'-wave area in lead aVL
ppareaaVR	P'-wave area in lead aVR
qampI	Q-wave amplitude in lead I
qampII	Q-wave amplitude in lead II
qampIII	Q-wave amplitude in lead III
qampV1	Q-wave amplitude in lead V1
qampV2	Q-wave amplitude in lead V2
qampV3	Q-wave amplitude in lead V3
qampV4	Q-wave amplitude in lead V4
qampV5	Q-wave amplitude in lead V5
qampV6	Q-wave amplitude in lead V6
qampaVF	Q-wave amplitude in lead aVF
qampaVL	Q-wave amplitude in lead aVL
qampaVR	Q-wave amplitude in lead aVR
qdurI	Q-wave duration in lead I
qdurII	Q-wave duration in lead II
qdurIII	Q-wave duration in lead III
qdurV1	Q-wave duration in lead V1
qdurV2	Q-wave duration in lead V2
qdurV3	Q-wave duration in lead V3
qdurV4	Q-wave duration in lead V4
qdurV5	Q-wave duration in lead V5
qdurV6	Q-wave duration in lead V6
qduraVF	Q-wave duration in lead aVF
qduraVL	Q-wave duration in lead aVL
qduraVR	Q-wave duration in lead aVR
rampI	R-wave amplitude in lead I
rampII	R-wave amplitude in lead II
rampIII	R-wave amplitude in lead III
rampV1	R-wave amplitude in lead V1
rampV2	R-wave amplitude in lead V2
rampV3	R-wave amplitude in lead V3
rampV4	R-wave amplitude in lead V4
rampV5	R-wave amplitude in lead V5

rampV6	R-wave amplitude in lead V6
rampaVF	R-wave amplitude in lead aVF
rampaVL	R-wave amplitude in lead aVL
rampaVR	R-wave amplitude in lead aVR
rdurI	R-wave duration in lead I
rdurII	R-wave duration in lead II
rdurIII	R-wave duration in lead III
rdurV1	R-wave duration in lead V1
rdurV2	R-wave duration in lead V2
rdurV3	R-wave duration in lead V3
rdurV4	R-wave duration in lead V4
rdurV5	R-wave duration in lead V5
rdurV6	R-wave duration in lead V6
rduraVF	R-wave duration in lead aVF
rduraVL	R-wave duration in lead aVL
rduraVR	R-wave duration in lead aVR
sampI	S-wave amplitude in lead I
sampII	S-wave amplitude in lead II
sampIII	S-wave amplitude in lead III
sampV1	S-wave amplitude in lead V1
sampV2	S-wave amplitude in lead V2
sampV3	S-wave amplitude in lead V3
sampV4	S-wave amplitude in lead V4
sampV5	S-wave amplitude in lead V5
sampV6	S-wave amplitude in lead V6
sampaVF	S-wave amplitude in lead aVF
sampaVL	S-wave amplitude in lead aVL
sampaVR	S-wave amplitude in lead aVR
sdurI	S-wave duration in lead I
sdurII	S-wave duration in lead II
sdurIII	S-wave duration in lead III
sdurV1	S-wave duration in lead V1
sdurV2	S-wave duration in lead V2
sdurV3	S-wave duration in lead V3
sdurV4	S-wave duration in lead V4
sdurV5	S-wave duration in lead V5
sdurV6	S-wave duration in lead V6
sduraVF	S-wave duration in lead aVF
sduraVL	S-wave duration in lead aVL
sduraVR	S-wave duration in lead aVR
rpampI	R'-wave amplitude in lead I
rpampII	R'-wave amplitude in lead II
rpampIII	R'-wave amplitude in lead III
rpampV1	R'-wave amplitude in lead V1
rpampV2	R'-wave amplitude in lead V2
rpampV3	R'-wave amplitude in lead V3

rpampV4	R'-wave amplitude in lead V4
rpampV5	R'-wave amplitude in lead V5
rpampV6	R'-wave amplitude in lead V6
rpampaVF	R'-wave amplitude in lead aVF
rpampaVL	R'-wave amplitude in lead aVL
rpampaVR	R'-wave amplitude in lead aVR
rpdurI	R'-wave duration in lead I
rpdurII	R'-wave duration in lead II
rpdurIII	R'-wave duration in lead III
rpdurV1	R'-wave duration in lead V1
rpdurV2	R'-wave duration in lead V2
rpdurV3	R'-wave duration in lead V3
rpdurV4	R'-wave duration in lead V4
rpdurV5	R'-wave duration in lead V5
rpdurV6	R'-wave duration in lead V6
rpduraVF	R'-wave duration in lead aVF
rpduraVL	R'-wave duration in lead aVL
rpduraVR	R'-wave duration in lead aVR
spampI	S'-wave amplitude in lead I
spampII	S'-wave amplitude in lead II
spampIII	S'-wave amplitude in lead III
spampV1	S'-wave amplitude in lead V1
spampV2	S'-wave amplitude in lead V2
spampV3	S'-wave amplitude in lead V3
spampV4	S'-wave amplitude in lead V4
spampV5	S'-wave amplitude in lead V5
spampV6	S'-wave amplitude in lead V6
spampaVF	S'-wave amplitude in lead aVF
spampaVL	S'-wave amplitude in lead aVL
spampaVR	S'-wave amplitude in lead aVR
spdurI	S'-wave duration in lead I
spdurII	S'-wave duration in lead II
spdurIII	S'-wave duration in lead III
spdurV1	S'-wave duration in lead V1
spdurV2	S'-wave duration in lead V2
spdurV3	S'-wave duration in lead V3
spdurV4	S'-wave duration in lead V4
spdurV5	S'-wave duration in lead V5
spdurV6	S'-wave duration in lead V6
spduraVF	S'-wave duration in lead aVF
spduraVL	S'-wave duration in lead aVL
spduraVR	S'-wave duration in lead aVR
vatI	Ventricular Activation Time in lead I
vatII	Ventricular Activation Time in lead II
vatIII	Ventricular Activation Time in lead III
vatV1	Ventricular Activation Time in lead V1

vatV2	Ventricular Activation Time in lead V2
vatV3	Ventricular Activation Time in lead V3
vatV4	Ventricular Activation Time in lead V4
vatV5	Ventricular Activation Time in lead V5
vatV6	Ventricular Activation Time in lead V6
vataVF	Ventricular Activation Time in lead aVF
vataVL	Ventricular Activation Time in lead aVL
vataVR	Ventricular Activation Time in lead aVR
qrsppkI	Peak-to-peak QRS complex amplitude in lead I
qrsppkII	Peak-to-peak QRS complex amplitude in lead II
qrsppkIII	Peak-to-peak QRS complex amplitude in lead III
qrsppkV1	Peak-to-peak QRS complex amplitude in lead V1
qrsppkV2	Peak-to-peak QRS complex amplitude in lead V2
qrsppkV3	Peak-to-peak QRS complex amplitude in lead V3
qrsppkV4	Peak-to-peak QRS complex amplitude in lead V4
qrsppkV5	Peak-to-peak QRS complex amplitude in lead V5
qrsppkV6	Peak-to-peak QRS complex amplitude in lead V6
qrsppkaVF	Peak-to-peak QRS complex amplitude in lead aVF
qrsppkaVL	Peak-to-peak QRS complex amplitude in lead aVL
qrsppkaVR	Peak-to-peak QRS complex amplitude in lead aVR
qrsdurI	QRS duration in lead I
qrsdurII	QRS duration in lead II
qrsdurIII	QRS duration in lead III
qrsdurV1	QRS duration in lead V1
qrsdurV2	QRS duration in lead V2
qrsdurV3	QRS duration in lead V3
qrsdurV4	QRS duration in lead V4
qrsdurV5	QRS duration in lead V5
qrsdurV6	QRS duration in lead V6
qrsduraVF	QRS duration in lead aVF
qrsduraVL	QRS duration in lead aVL
qrsduraVR	QRS duration in lead aVR
qrsareaI	QRS area in lead I
qrsareaII	QRS area in lead II
qrsareaIII	QRS area in lead III
qrsareaV1	QRS area in lead V1
qrsareaV2	QRS area in lead V2
qrsareaV3	QRS area in lead V3
qrsareaV4	QRS area in lead V4
qrsareaV5	QRS area in lead V5
qrsareaV6	QRS area in lead V6
qrsareaaVF	QRS area in lead aVF
qrsareaaVL	QRS area in lead aVL
qrsareaaVR	QRS area in lead aVR
stonI	ST onset in lead I
stonII	ST onset in lead II

stonIII	ST onset in lead III
stonV1	ST onset in lead V1
stonV2	ST onset in lead V2
stonV3	ST onset in lead V3
stonV4	ST onset in lead V4
stonV5	ST onset in lead V5
stonV6	ST onset in lead V6
stonaVF	ST onset in lead aVF
stonaVL	ST onset in lead aVL
stonaVR	ST onset in lead aVR
stmidI	ST mid-segment in lead I
stmidII	ST mid-segment in lead II
stmidIII	ST mid-segment in lead III
stmidV1	ST mid-segment in lead V1
stmidV2	ST mid-segment in lead V2
stmidV3	ST mid-segment in lead V3
stmidV4	ST mid-segment in lead V4
stmidV5	ST mid-segment in lead V5
stmidV6	ST mid-segment in lead V6
stmidaVF	ST mid-segment in lead aVF
stmidaVL	ST mid-segment in lead aVL
stmidaVR	ST mid-segment in lead aVR
st80I	ST segment 80 ms after onset in lead I
st80II	ST segment 80 ms after onset in lead II
st80III	ST segment 80 ms after onset in lead III
st80V1	ST segment 80 ms after onset in lead V1
st80V2	ST segment 80 ms after onset in lead V2
st80V3	ST segment 80 ms after onset in lead V3
st80V4	ST segment 80 ms after onset in lead V4
st80V5	ST segment 80 ms after onset in lead V5
st80V6	ST segment 80 ms after onset in lead V6
st80aVF	ST segment 80 ms after onset in lead aVF
st80aVL	ST segment 80 ms after onset in lead aVL
st80aVR	ST segment 80 ms after onset in lead aVR
stendI	ST end in lead I
stendII	ST end in lead II
stendIII	ST end in lead III
stendV1	ST end in lead V1
stendV2	ST end in lead V2
stendV3	ST end in lead V3
stendV4	ST end in lead V4
stendV5	ST end in lead V5
stendV6	ST end in lead V6
stendaVF	ST end in lead aVF
stendaVL	ST end in lead aVL
stendaVR	ST end in lead aVR

stdurI	ST duration in lead I
stdurII	ST duration in lead II
stdurIII	ST duration in lead III
stdurV1	ST duration in lead V1
stdurV2	ST duration in lead V2
stdurV3	ST duration in lead V3
stdurV4	ST duration in lead V4
stdurV5	ST duration in lead V5
stdurV6	ST duration in lead V6
stduraVF	ST duration in lead aVF
stduraVL	ST duration in lead aVL
stduraVR	ST duration in lead aVR
stslopeI	ST slope in lead I
stslopeII	ST slope in lead II
stslopeIII	ST slope in lead III
stslopeV1	ST slope in lead V1
stslopeV2	ST slope in lead V2
stslopeV3	ST slope in lead V3
stslopeV4	ST slope in lead V4
stslopeV5	ST slope in lead V5
stslopeV6	ST slope in lead V6
stslopeaVF	ST slope in lead aVF
stslopeaVL	ST slope in lead aVL
stslopeaVR	ST slope in lead aVR
tampI	T-wave amplitude in lead I
tampII	T-wave amplitude in lead II
tampIII	T-wave amplitude in lead III
tampV1	T-wave amplitude in lead V1
tampV2	T-wave amplitude in lead V2
tampV3	T-wave amplitude in lead V3
tampV4	T-wave amplitude in lead V4
tampV5	T-wave amplitude in lead V5
tampV6	T-wave amplitude in lead V6
tampaVF	T-wave amplitude in lead aVF
tampaVL	T-wave amplitude in lead aVL
tampaVR	T-wave amplitude in lead aVR
tdurI	T-wave duration in lead I
tdurII	T-wave duration in lead II
tdurIII	T-wave duration in lead III
tdurV1	T-wave duration in lead V1
tdurV2	T-wave duration in lead V2
tdurV3	T-wave duration in lead V3
tdurV4	T-wave duration in lead V4
tdurV5	T-wave duration in lead V5
tdurV6	T-wave duration in lead V6
tduraVF	T-wave duration in lead aVF

tduraVL	T-wave duration in lead aVL
tduraVR	T-wave duration in lead aVR
tareaI	T-wave area in lead I
tareaII	T-wave area in lead II
tareaIII	T-wave area in lead III
tareaV1	T-wave area in lead V1
tareaV2	T-wave area in lead V2
tareaV3	T-wave area in lead V3
tareaV4	T-wave area in lead V4
tareaV5	T-wave area in lead V5
tareaV6	T-wave area in lead V6
tareaaVF	T-wave area in lead aVF
tareaaVL	T-wave area in lead aVL
tareaaVR	T-wave area in lead aVR
tpampI	T'-wave amplitude in lead I
tpampII	T'-wave amplitude in lead II
tpampIII	T'-wave amplitude in lead III
tpampV1	T'-wave amplitude in lead V1
tpampV2	T'-wave amplitude in lead V2
tpampV3	T'-wave amplitude in lead V3
tpampV4	T'-wave amplitude in lead V4
tpampV5	T'-wave amplitude in lead V5
tpampV6	T'-wave amplitude in lead V6
tpampaVF	T'-wave amplitude in lead aVF
tpampaVL	T'-wave amplitude in lead aVL
tpampaVR	T'-wave amplitude in lead aVR
tpdurI	T'-wave duration in lead I
tpdurII	T'-wave duration in lead II
tpdurIII	T'-wave duration in lead III
tpdurV1	T'-wave duration in lead V1
tpdurV2	T'-wave duration in lead V2
tpdurV3	T'-wave duration in lead V3
tpdurV4	T'-wave duration in lead V4
tpdurV5	T'-wave duration in lead V5
tpdurV6	T'-wave duration in lead V6
tpduraVF	T'-wave duration in lead aVF
tpduraVL	T'-wave duration in lead aVL
tpduraVR	T'-wave duration in lead aVR
tpareaI	T'-wave area in lead I
tpareaII	T'-wave area in lead II
tpareaIII	T'-wave area in lead III
tpareaV1	T'-wave area in lead V1
tpareaV2	T'-wave area in lead V2
tpareaV3	T'-wave area in lead V3
tpareaV4	T'-wave area in lead V4
tpareaV5	T'-wave area in lead V5

tpareaV6	T'-wave area in lead V6
tpareaaVF	T'-wave area in lead aVF
tpareaaVL	T'-wave area in lead aVL
tpareaaVR	T'-wave area in lead aVR
printI	PR interval in lead I
printII	PR interval in lead II
printIII	PR interval in lead III
printV1	PR interval in lead V1
printV2	PR interval in lead V2
printV3	PR interval in lead V3
printV4	PR interval in lead V4
printV5	PR interval in lead V5
printV6	PR interval in lead V6
printaVF	PR interval in lead aVF
printaVL	PR interval in lead aVL
printaVR	PR interval in lead aVR
prsegI	PR segment in lead I
prsegII	PR segment in lead II
prsegIII	PR segment in lead III
prsegV1	PR segment in lead V1
prsegV2	PR segment in lead V2
prsegV3	PR segment in lead V3
prsegV4	PR segment in lead V4
prsegV5	PR segment in lead V5
prsegV6	PR segment in lead V6
prsegaVF	PR segment in lead aVF
prsegaVL	PR segment in lead aVL
prsegaVR	PR segment in lead aVR
qtintI	QT interval in lead I
qtintII	QT interval in lead II
qtintIII	QT interval in lead III
qtintV1	QT interval in lead V1
qtintV2	QT interval in lead V2
qtintV3	QT interval in lead V3
qtintV4	QT interval in lead V4
qtintV5	QT interval in lead V5
qtintV6	QT interval in lead V6
qtintaVF	QT interval in lead aVF
qtintaVL	QT interval in lead aVL
qtintaVR	QT interval in lead aVR

Table S2. Optimum cut-off values and area under the curve (AUC) from receiver operating characteristic (ROC) analysis of selected ECG variables (those with P-value<0.05).

Characteristics	Optimum cut-off	AUC	95% CI
Vector magnitude of the initial 40 ms transverse QRS signal	0.92	0.68	0.32-1.00
QRS horizontal axis	57.5	0.67	0.45-0.90
ST horizontal axis	65.5	0.30	0.12-0.48
R-wave amplitude in lead I	0.6715	0.36	0.19-0.53
R-wave duration in lead III	50	0.71	0.52-0.90
S-wave amplitude in lead I	-0.144	0.26	0.10-0.42
S-wave duration in lead aVL	35.5	0.86	0.72-1.00
QRS duration in lead V3	96.5	0.72	0.57-0.86
QRS area in lead I	0.75	0.21	0.09-0.32
ST slope in lead I	31.5	0.81	0.68-0.93
T-wave area in V1	-3.05	0.27	0.11-0.42
PR interval in V2	157	0.59	0.40-0.78

Table S3. Creation of a weighted score system.

Number	ECG variable	Beta	Beta Proportion	Points	P-value	1/(P value)	proportion of (1/(P-value))	Adjusted final points
1	Initial type 1 pattern	1.29	0.05	5	0.037	27	0.01	0.05
2	Initial syncope	1.66	0.07	7	0.044	23	0.01	0.06
3	Initial VT/VF	1.97	0.08	8	0.001	1000	0.37	3.00
4	QRS horizontal axis > 57.5 deg	1.34	0.06	6	0.023	43	0.02	0.09
5	ST horizontal axis < 65.5 deg	0.44	0.02	2	0.448	2	0.00	0.00
6	R-wave amplitude in lead I < 0.6715 mV	0.37	0.02	2	0.562	2	0.00	0.00
7	R-wave duration in lead III > 50 ms	1.57	0.06	6	0.019	53	0.02	0.13
8	S-wave amplitude in lead I < -0.144 mV	0.76	0.03	3	0.265	4	0.00	0.00
9	S-wave duration in lead aVL > 35.5 ms	2.95	0.12	12	0.005	200	0.07	0.90
10	QRS duration in V3 > 96.5 ms	1.20	0.05	5	0.075	13	0.00	0.02
11	QRS area in lead aVL > 0.75 Ashman units	5.73	0.24	24	0.005	200	0.07	1.75
12	ST slope in lead I > 31.5 deg	1.80	0.07	7	0.008	125	0.05	0.34
13	T-wave area in lead V1 < -3.05 Ashman units	0.79	0.03	3	0.178	6	0.00	0.01
14	PR interval in lead V2 > 157 ms	2.39	0.10	10	0.001	1000	0.37	3.65
	Sum	24.25	1	100	-	2698	-	10.01

Table S4. Final weighted score system.

Number	ECG variable	Beta	Beta Proportion	Points	P-value	1/(P value)	proportion of (1/(P-value))	Adjusted final points
1	Initial type 1 pattern	1.29	0.05	5	0.037	27	0.01	0.05
2	Initial syncope	1.66	0.07	7	0.044	23	0.01	0.06
3	Initial VT/VF	1.97	0.08	8	0.001	1000	0.37	3.00
4	QRS horizontal axis > 57.5 deg	1.34	0.06	6	0.023	43	0.02	0.09
5	R-wave duration in lead III > 50 ms	1.57	0.06	6	0.019	53	0.02	0.13
6	S-wave duration in lead aVL > 35.5 ms	2.95	0.12	12	0.005	200	0.07	0.90
7	QRS duration in V3 > 96.5 ms	1.20	0.05	5	0.075	13	0.00	0.02
8	QRS area in lead aVL > 0.75 Ashman units	5.73	0.24	24	0.005	200	0.07	1.75
9	ST slope in lead I > 31.5 deg	1.80	0.07	7	0.008	125	0.05	0.34
10	T-wave area in lead V1 < -3.05 Ashman units	0.79	0.03	3	0.178	6	0.00	0.01
11	PR interval in lead V2 > 157 ms	2.39	0.10	10	0.001	1000	0.37	3.65

Table S5. Weighted score system based on significant dichotomized ECG variables.

Characteristics	Cut-off	AUC (95% CI)	HR (95% CI)	P-value
Weighted score	4.275	0.84 (0.72-0.97)	1.59 (1.27-2.00)	**<0.0001**
Weighted score > 4.275	-	0.88 (0.68-0.95)	14.88 (3.99-55.50)	**<0.0001**

Table S6. Characteristics differences among the variables to create a weighted score system. *for SMD≤0.2.

Characteristics	Next VT (N=12) Mean(SD);Max;N or Count(%)	No next VT (N=71) Mean(SD);Max;N or Count(%)	SMD
Initial type 1 pattern	8(66.66%)	29(40.84%)	0.54
Initial syncope	6(50.00%)	23(32.39%)	0.36
Initial VT/VF	5(41.66%)	4(5.63%)	0.94
QRS horizontal axis > 57.5 deg	7(58.33%)	13(18.30%)	0.9
ST horizontal axis < 65.5 deg	6(50.00%)	28(39.43%)	0.21
R-wave amplitude in lead I < 0.6715 mV	9(75.00%)	46(64.78%)	0.22
rampi06715	8(66.66%)	48(67.60%)	0.02*
R-wave duration in lead III > 50 ms	9(75.00%)	26(36.61%)	0.84
S-wave amplitude in lead I < -0.144 mV	9(75.00%)	35(49.29%)	0.55
S-wave duration in lead aVL > 35.5 ms	11(91.66%)	19(26.76%)	1.76
QRS duration in V3 > 96.5 ms	9(75.00%)	26(36.61%)	0.84
QRS area in lead aVL > 0.75 Ashman units	4(33.33%)	3(4.22%)	0.8
ST slope in lead I > 31.5 deg	9(75.00%)	14(19.71%)	1.33
T-wave area in lead V1 < -3.05 Ashman units	7(58.33%)	29(40.84%)	0.36
PR interval in lead V2 > 157 ms	8(66.66%)	33(46.47%)	0.42

Table S7. Confusion matrix of using decision tree learning model to predict next VT.

	Predicted Not Next VT	Predicted Next VT
True Not Next VT	68	3
True Next VT	0	12

Supplementary Figures

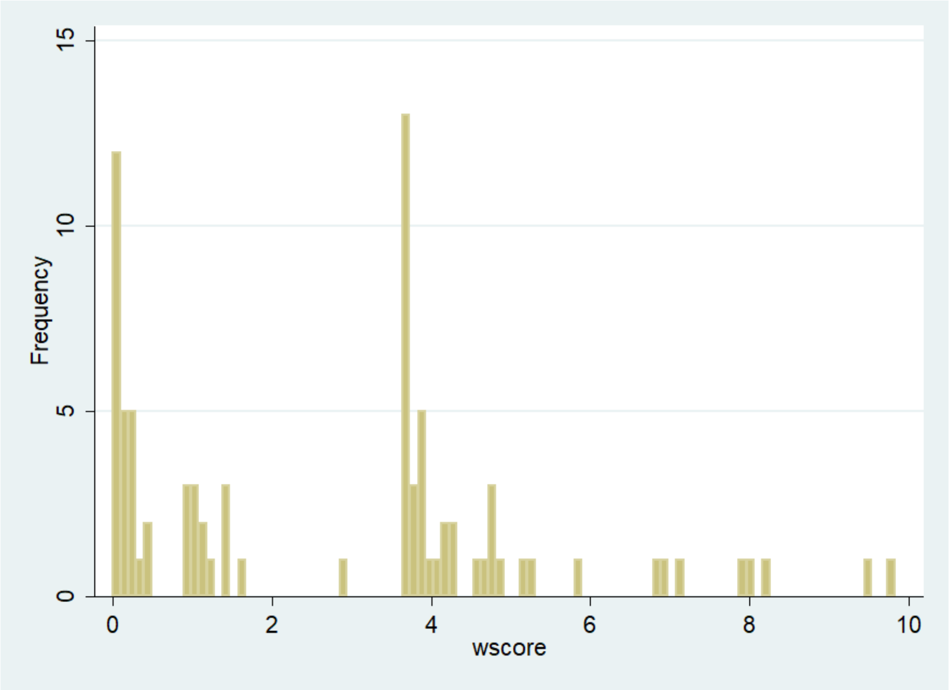

Figure S1. Histogram of frequency against the weighted score.

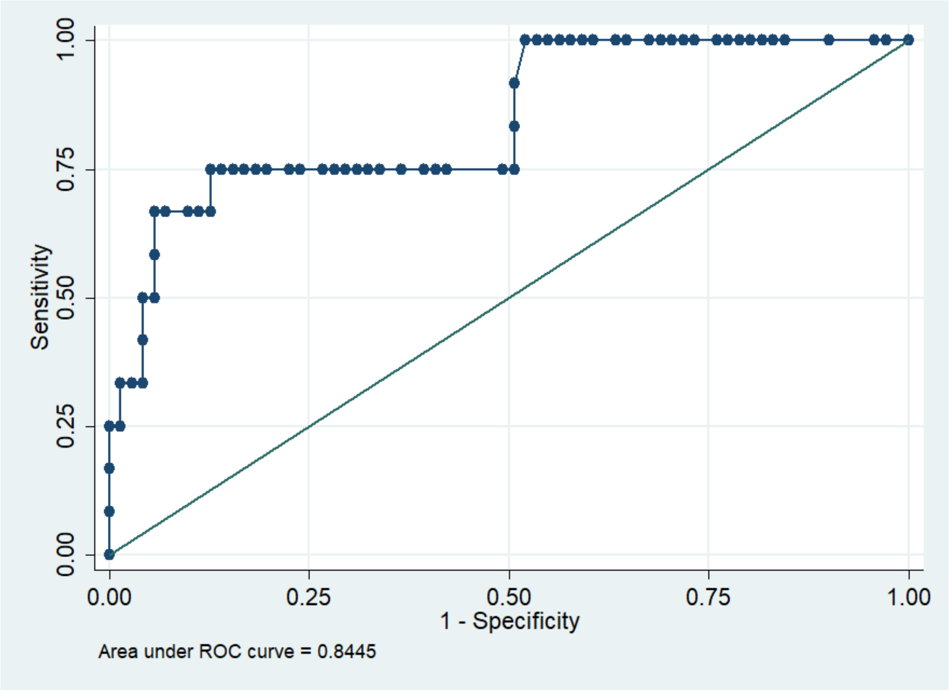

Figure S2. Receiver operating characteristics (ROC) analysis of the weighted score for predicting incident spontaneous VT/VF.

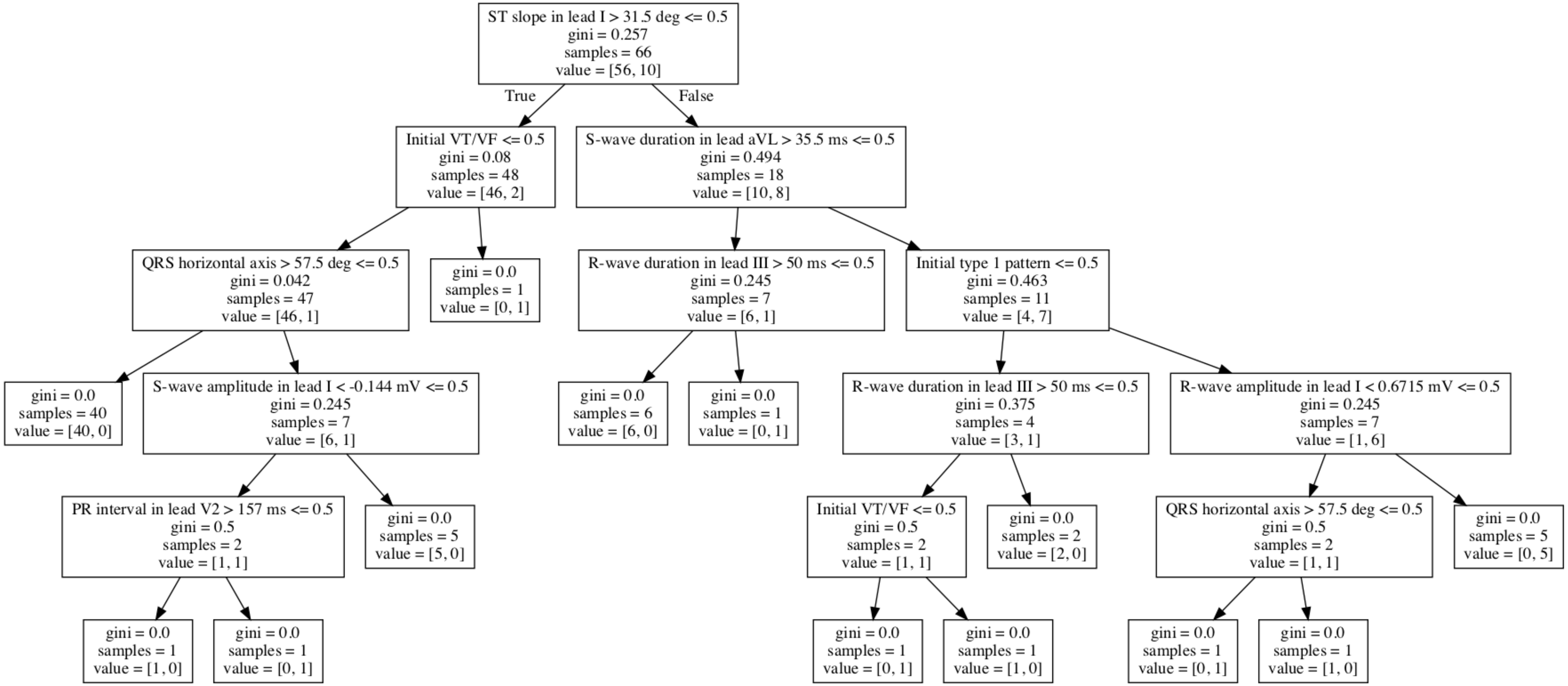

Figure S3. Decision rules generated by the decision tree learning model (Note: here '<=0.5' indicates the variables takes 0).
