# Supplementary material for: Automated Electrocardiogram Analysis Identifies Novel Predictors of Ventricular Arrhythmias in Brugada Syndrome
Source: Front Cardiovasc Med. 2021 Jan 14;7:618254. doi: 10.3389/fcvm.2020.618254 (PMC7840575; doi:10.3389/fcvm.2020.618254)
Supplement: Supplementary file 2 [file Presentation_1.PPTX]

## Slide 1
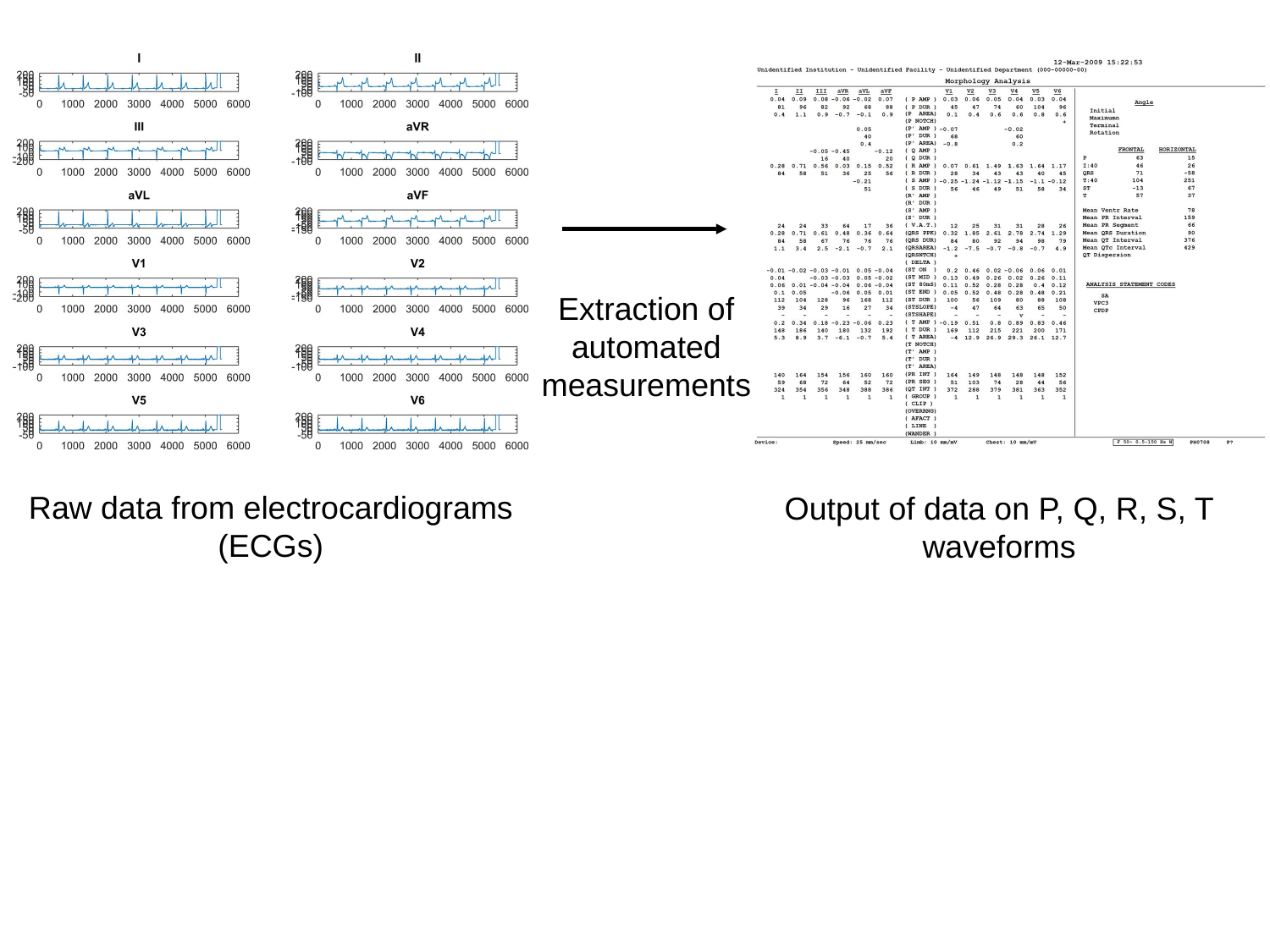

Extraction of automated measurements
Raw data from electrocardiograms (ECGs)
Output of data on P, Q, R, S, T waveforms

## Slide 2
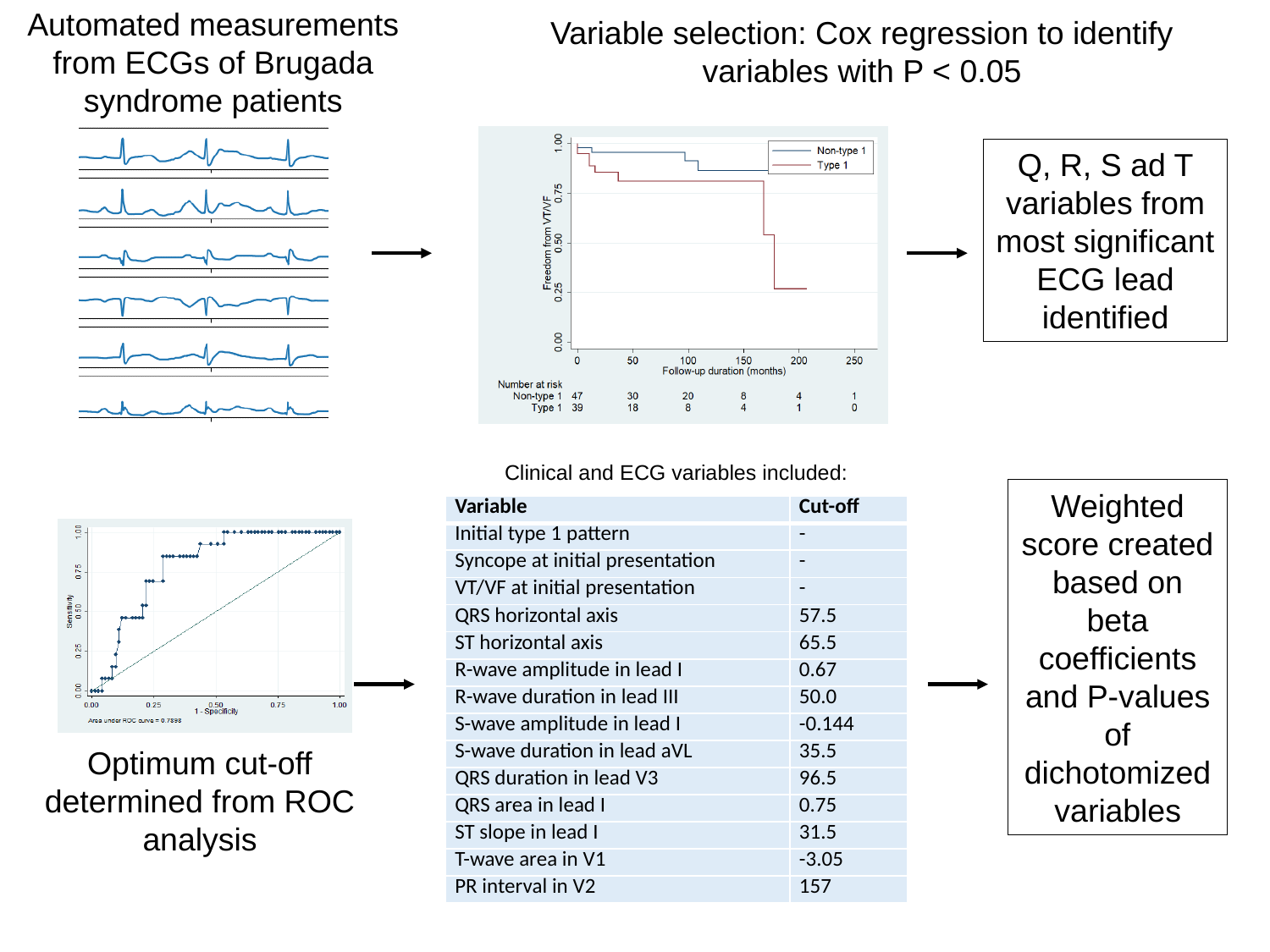

Automated measurements from ECGs of Brugada syndrome patients
Variable selection: Cox regression to identify variables with P < 0.05
Q, R, S ad T variables from most significant ECG lead identified
Clinical and ECG variables included:
Weighted score created based on beta coefficients and P-values of dichotomized variables
| Variable | Cut-off |
| --- | --- |
| Initial type 1 pattern | - |
| Syncope at initial presentation | - |
| VT/VF at initial presentation | - |
| QRS horizontal axis | 57.5 |
| ST horizontal axis | 65.5 |
| R-wave amplitude in lead I | 0.67 |
| R-wave duration in lead III | 50.0 |
| S-wave amplitude in lead I | -0.144 |
| S-wave duration in lead aVL | 35.5 |
| QRS duration in lead V3 | 96.5 |
| QRS area in lead I | 0.75 |
| ST slope in lead I | 31.5 |
| T-wave area in V1 | -3.05 |
| PR interval in V2 | 157 |
Optimum cut-off determined from ROC analysis
